# Data Augmentation with Cross-Modal Variational Autoencoders (DACMVA) for Cancer Survival Prediction

**DOI:** 10.3390/info15010007

**Published:** 2023-12-21

**Authors:** Sara Rajaram, Cassie S. Mitchell

**Affiliations:** 1 Laboratory for Pathology Dynamics, Georgia Institute of Technology and Emory University, Atlanta, GA 30332, USA; 2 Center for Machine Learning at Georgia Tech, Georgia Institute of Technology, Atlanta, GA 30332, USA

**Keywords:** data augmentation, Variational Autoencoder, Generative Adversarial Network, cancer survival prediction

## Abstract

The ability to translate Generative Adversarial Networks (GANs) and Variational Autoencoders (VAEs) into different modalities and data types is essential to improve Deep Learning (DL) for predictive medicine. This work presents DACMVA, a novel framework to conduct data augmentation in a cross-modal dataset by translating between modalities and oversampling imputations of missing data. DACMVA was inspired by previous work on the alignment of latent spaces in Autoencoders. DACMVA is a DL data augmentation pipeline that improves the performance in a downstream prediction task. The unique DACMVA framework leverages a cross-modal loss to improve the imputation quality and employs training strategies to enable regularized latent spaces. Oversampling of augmented data is integrated into the prediction training. It is empirically demonstrated that the new DACMVA framework is effective in the often-neglected scenario of DL training on tabular data with continuous labels. Specifically, DACMVA is applied towards cancer survival prediction on tabular gene expression data where there is a portion of missing data in a given modality. DACMVA significantly (*p* << 0.001, one-sided Wilcoxon signed-rank test) outperformed the non-augmented baseline and competing augmentation methods with varying percentages of missing data (4%, 90%, 95% missing). As such, DACMVA provides significant performance improvements, even in very-low-data regimes, over existing state-of-the-art methods, including TDImpute and oversampling alone.

## 1. Introduction

Deep Learning (DL) has proven to be fruitful for use in biomedical prediction tasks, but the risk of overfitting remains due to the limited size of such datasets. To mitigate overfitting in such low-data regimes, researchers often synthesize data using generative DL algorithms, such as Generative Adversarial Networks (GANs) and Variational Autoencoders (VAEs) [1,2]. However, the majority of such applications are geared towards classification tasks. For example, Chen et al. [3] reviews image synthesis for medical prediction tasks and notes that most studies train a GAN for each class. Such methods are not directly applicable to ML regression tasks, but nonetheless indicate the utility of GANs for data augmentation.

Likewise, researchers have demonstrated success in biomedical tasks with VAEs. For example, Doncevic and Herrmann [4] developed a VAE architecture with an interpretable latent space and decoder for medical application. It enabled the perturbation of input features to understand changes in the activation of hidden nodes. By doing so, it simulates the effects of genetic changes on a resulting phenotype as well as the drug response predictions of models [4]. Likewise, Papadopoulos and Karalis [5] employed a VAE framework to synthesize clinical study patient samples. Their results showed that including the synthetic data provides greater statistical power than using the original dataset alone [5].

Biomedical datasets often contain multiple modalities, such as genomics, imaging, clinician notes, peripheral blood tests, audio recordings, and more. A major limitation of current multi-modal models is that they often cannot make full use of missing data, namely, missing modalities [6]. Historically, predictive medicine models would simply discard records that did not have all of the desired modalities. Disregarding missing modalities or records drastically reduces the available sample size, which decreases the quality of the predictions and the generalizability of the model.

Novel DL methods for modality translation on medical datasets exist in the literature, but such methods are highly task-specific towards imaging modalities. As discussed by Armanious et al. [7], most methods focus on translating between computed tomography (CT), magnetic resonance imaging (MRI), and positron emission tomography (PET). In addition to being limited to the types of imaging, they also employ specialized architectures to account for the motion or jitter often seen in medical imaging. CycleGAN is another popular method for image-to-image translation. CycleGAN involves the training of two generators and two discriminators with a cycle consistency loss [8]. Sandfort et al. [9] successfully applied CycleGAN to transform contrast CT images into non-contrast CT images and used the augmented dataset to improve the segmentation performance. However, CycleGAN is primarily beneficial for color or texture type transformations. It was originally developed to translate between two views of the same modality. Therefore, existing imaging-centric methods, including MedGan [7] and CycleGAN [8], are not directly applicable to the important case of tabular data modalities that carry distinct information.

Recently, Yang et al. [10] proposed a model that substantially surpasses CycleGAN to align single cell RNA-seq and ATAC-seq data. They trained Autoencoders (AE) for the modalities of interest, and they aligned the AE latent spaces through adversarial training with an additional discriminator network. Then, they paired the modality A encoder and modality B decoder to align single-cell data. Zhou et al. [11] trained an encoder of modality A along with the decoder of modality B for imputation in the cancer survival prediction task. However, these works did not consider stable training methods to regularize the Autoencoder latent spaces or the integrated oversampling of synthetic data.

The ability to translate GANs and VAEs into different modalities and data types is essential to improve DL for predictive medicine. Here, we present data augmentation for Cross-Modal Variational Autoencoders (DACMVA), which builds upon the aforementioned related works by incorporating VAEs to translate between different data modalities, including the critically important, but often neglected, tabular data types common in medicine. Specifically, DACMVA takes advantage of modality A to impute samples of modality B and vice versa. Such cross-modal imputations are particularly advantageous in the case of a large imbalance in the sample counts between the two modalities. In addition, DACMVA can carry over the outcome value associated with the modality A sample to the imputed modality B sample to circumvent the issue of imputing a continuous label. DACMVA demonstrates that regularized latent spaces in VAEs result in an improved imputation quality over deterministic AEs. Additionally, prior related works have not integrated oversampling. The inclusion of oversampling is another benefit of DACMVA.

In summary, this work presents DACMVA, a novel DL pipeline for data augmentation with Cross-Modal Variational Autoencoders. DACMVA demonstrates a superior performance in the task of cancer survival prediction using tabular gene expression data. The key contributions of this study are the following:

- DACMVA proposes a pipeline for training Variational Autoencoders (VAEs) for modalities A and B with aligned latent spaces. It incorporate strategies to improve the stability during VAE training, thereby enabling regularized latent spaces.
- DACMVA oversamples the imputed samples with hyperparameters to control the imputed batch size; a loss threshold for selecting imputed samples; and a weight for the loss on the imputed batches. This flexible and tunable framework integrates with the cross-modal imputation method for simple, but effective, oversampling augmentation.
- The role of the adversarial training strategy proposed by Yang et al. [10] for aligning the latent spaces is empirically investigated. In particular, we determine whether the augmentations created by Autoencoders trained adversarially result in a significantly improved performance in the prediction task. Adversarial training comes with an additional computational cost and training stability challenges which may be infeasible for large, high-dimensional datasets. Thus, this analysis is informative for many applications.
- The novel DACMVA framework was applied over multiple augmentation methods for cancer survival prediction. To our knowledge, this study is the first to investigate the roles of oversampling and adversarial loss in data augmentation in cancer survival prediction. The results illustrate the ability of DACMVA to improve model predictions on multi-modal tabular biomedical data with continuous labels. The results show that the presented DACMVA framework generates high-quality imputations and provides a significantly improved task performance with both the full dataset and in low-data regimes.

The presentation of DACMVA and its application to cancer survival predictions is organized as follows: Section 2 introduces an overview of the DACMVA framework and provides the details of the methodology and the training procedure. Section 3 presents the results for the imputation quality and the performance in the multi-modal cancer survival prediction task using the original dataset and the low-data regime setting. Finally, the conclusions and future directions for DACMVA are summarized in Section 4.

## 2. Materials and Methods

Here, the DACMVA framework is presented. The primary goal of DACMVA is to improve the model’s predictive performance on multi-modal, tabular biomedical datasets, including low-data regimes. DACMVA stands for data augmentation with Cross-Modal Variational Autoencoders. The DACMVA framework includes Cross-Modal Variational Autoencoders with adversarial training (CM-VAE-Adv); Cross-Modal Variational Autoencoders with oversampling (CM-VAE-OV); and the combination of adversarial training and oversampling (CM-VAE-Adv-OV).

### 2.1. Overview, Nomenclature, and Introductory Definitions

**Figure 1.**
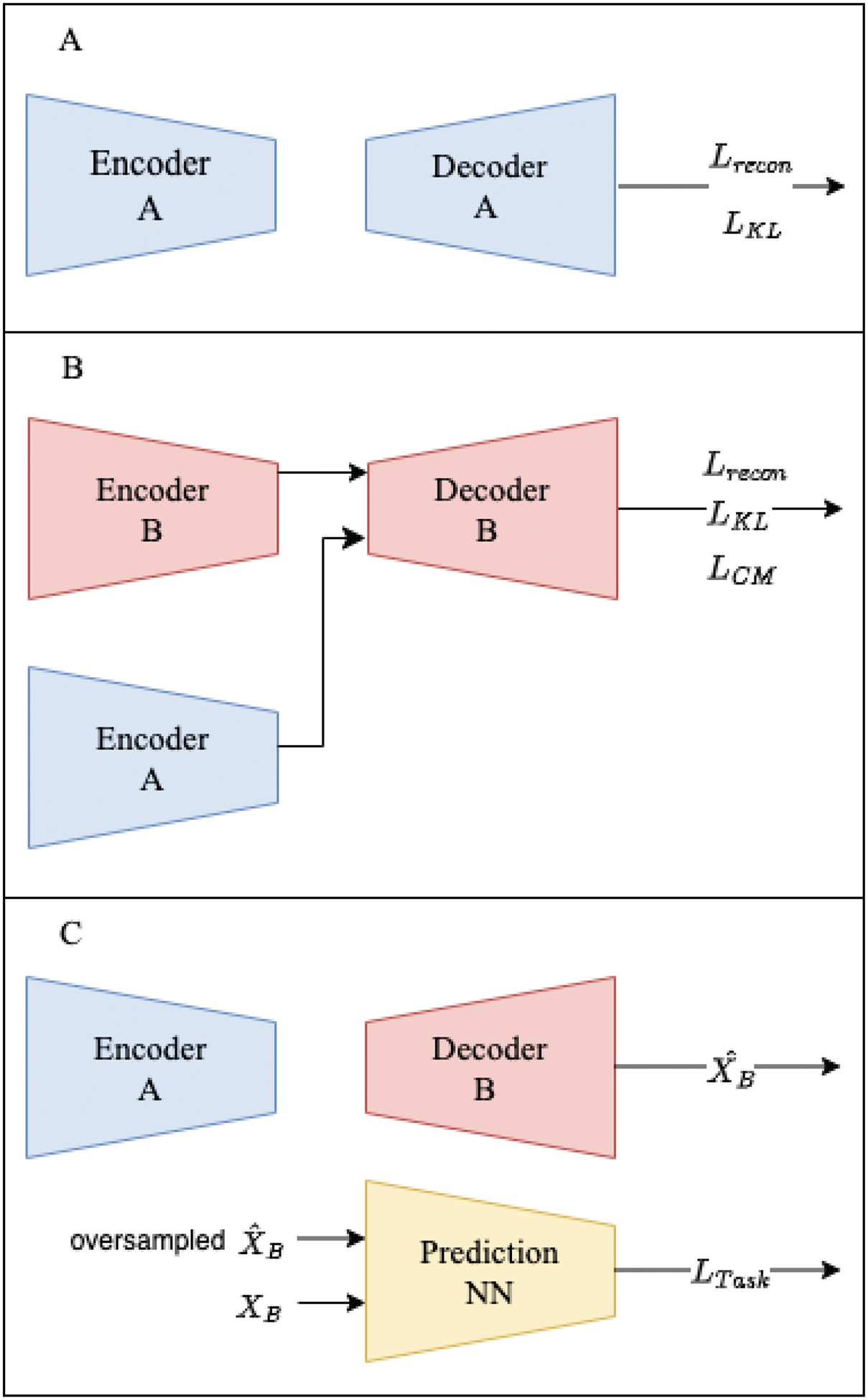
DACMVA training procedure for CM-VAE-OV. (**A**) Train the modality A VAE. (**B**) Train the modality B VAE, aligned with the A latent space. (**C**) Impute ${\hat{X}}_{B}$ and oversample for the training prediction task.

**Figure 2.**
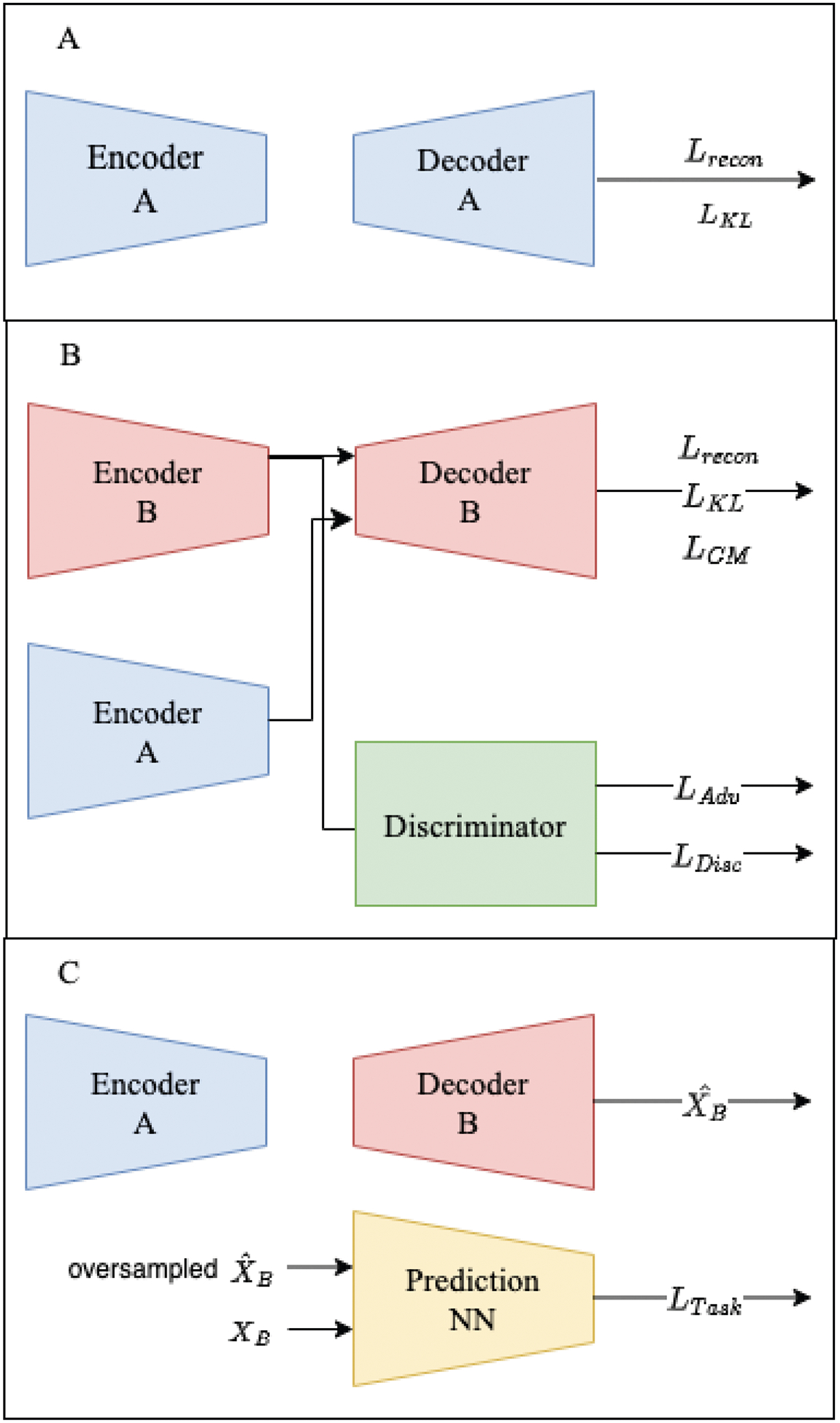
DACMVA training procedure for CM-VAE-Adv-OV. (**A**) Train the modality A VAE. (**B**) Train the modality B VAE, aligned with the A latent space, and train the discriminator to encourage alignment. (**C**) Impute ${\hat{X}}_{B}$ and oversample for the training prediction task.

The DACMVA framework was constructed to enable data augmentation with Cross-Modal Variational Autoencoders. Let us first define modalities A and B with their corresponding feature space inputs **X**_**A**_ and **X**_**B**_. We experiment with multiple variations of training, which are labeled CM-VAE-Adv, CM-VAE-OV, and CM-VAE-Adv-OV. Figure 1 depicts the CM-VAE-OV training, which excludes any adversarial training but includes oversampling of the synthetic data. Figure 2 provides the CM-VAE-Adv-OV training procedure, which includes both adversarial training and the oversampling of synthetic data. A figure for the CM-VAE-Adv training is not shown because it is identical to Figure 2 with the oversampling of ${\hat{\text{X}}}_{\text{B}}$ in part C omitted. In all variations, we train the modality A VAE as detailed in part A of the figures, and then we freeze this network for all subsequent steps. Conceptually, this choice facilitates the alignment of the modality B latent space to the static latent distribution provided by the modality A VAE. Additionally, reduced computational demand for tuning hyperparameters (HPs) is acquired to optimize the modality B VAE once we have a fixed VAE for modality A.

### 2.2. CM-VAE-Adv

The work presented by Makhzani et al. [12] introduced the concept of the adversarial AE to train an AE such that its latent space is matched to a prior distribution. Training proceeds in a GAN analogous framework. That is, a discriminator is optimized to distinguish between a sample from the prior and a sample from the latent space of the AE. The encoder of the AE is optimized to deceive the discriminator, while the AE also aims to minimize the reconstruction loss. Yang et al. [10] extends this concept towards the training of Autoencoders of different modalities adversarially in order to align their latent spaces.

Similarly, we construct AEs for modalities A and B as well as a discriminator neural network to distinguish samples from the latent spaces of the two modalities. In each epoch of training, we optimize the reconstruction loss, the Kullback–Leibler (KL) divergence loss, the discriminator loss, and an adversarial loss. Although we could use the traditional binary cross entropy losses for the discriminator and adversarial losses, we found an improved training stability when we used the least squares losses. The losses for this training framework are given by the following equations. The variables *z*_*A*_ and *z*_*B*_ refer to the latent representations produced from *Enc*_*A*_ and *Enc*_*B*_ respectively.


(1) $$L_{\text{recon}}=\sum_{i=A,B}\left|X_{i}-{\hat{X}}_{i}\right|\;L_{adv}={\left|\text{Disc}\left(z_{A}\right)-1\right|}^{2}+{\left|\text{Disc}\left(z_{B}\right)-0\right|}^{2}\;L_{\text{Disc}}={\left|\text{Disc}\left(z_{A}\right)-0\right|}^{2}+{\left|\text{Disc}\left(z_{B}\right)-1\right|}^{2}$$

Yang et al. [10] trained their AEs as deterministic or as VAEs with very small weights on the KL term, presumably due to the challenges of training high-dimensional datasets of limited size as VAEs. In such scenarios, the KL loss can vanish to zero, even with small but nonzero weights. We mitigate this vanishing loss by implementing a cyclic KL annealing schedule [13]. For our application, we consider it important to train the AEs as VAEs, because we aim to produce meaningful synthetic samples of one modality based on the latent representation of the other modality. If the latent space is not regularized, we can produce a non-meaningful imputation if a substantially original input is presented.

Next, we include a cross-modal reconstruction loss similarly to Zhou et al. [11]. We found that the inclusion of this loss improves the Pearson’s correlation coefficient between a test set of original samples and imputed samples for modality B. Our stopping condition for training the modality B VAE is to minimize this loss. We define the cross-modal loss, *L*_*cm*_, as follows:

(2) $$L_{CM}=\underset{i}{\sum }\left|X_{B,i}-Dec_{B}\left(Enc_{A}\left(X_{A,i}\right)\right)\right|$$

Next, we iterate through all *X*_*A*_ for which there is not a corresponding *X*_*B*_. For this missing data, we compute ${\hat{X}}_{B}$ as follows: ${\hat{X}}_{\text{B}}=Dec_{B}\left(Enc_{A}\left(X_{A}\right)\right)$ We then train the prediction task on a shuffled dataset of **X**_**B**_ and ${\hat{X}}_{\text{B}}$ We train the prediction task on modality B, only as a simplifying assumption and to examine the effects of augmentation for a single modality. As noted, we do not impute the continuous labels, but rather, carry them over from the corresponding *X*_*A*_.

### 2.3. CM-VAE-OV

The training schematic is provided in Figure 1. We train the modality B VAE with *L*_*recon*_, the KL divergence term, and the cross-modal loss (*L*_*CM*_). We do not have a discriminator nor the associated *L*_*adv*_ and *L*_*Disc*_ losses. This method relies solely on *L*_*CM*_ for latent space alignment.

Lastly, we train the prediction task with the original modality B training set and the oversampled synthetic data from the trained *Enc*_*A*_ and *Dec*_*B*_. In each epoch, we randomly sample with replacements from the modality A dataset, and we compute we compute the corresponding ${\hat{X}}_{B}$ We also compute the losses specific to the prediction task, *L*_*task*_, on these synthetic samples. Similar to Haque [14], we keep only those ${\hat{X}}_{B}$ with a task loss below a HP threshold, *t*. We additionally introduce the HP *bsOV* regulating the batch size of the ${\hat{X}}_{B}$ set. The prediction NN loss function on the ${\hat{X}}_{B}$ samples is weighted by a factor of 0 ≤ *γ*≤ 1

### 2.4. CM-VAE-Adv-Ov

This training variation uses all of the elements described in the previous subsections as well as those depicted in Figure 2. As presented in Section 2.2, we train the modality B VAE using *L*_*recon*_ and *L*_*KL*_ to produce high quality reconstructions and a regularized latent space. We also employ the discriminator and train with *L*_*adv*_ and *L*_*Disc*_ to encourage aligned latent spaces. As in the other variations, we have *L*_*CM*_ to additionally enforce alignment.

Training the prediction NN involves the oversampling procedure described in Section 2.3. Specifically, we oversample with replacements from the modality A dataset to produce synthetic modality B samples. We use the task specific loss threshold, *t*, to keep synthetic samples. We also have the HPs *bsOV* and *γ* controlling for the batch size and the weight on the task-specific loss for imputed samples.

### 2.5. Cancer Survival Prediction Training Details

The utility of the above-described new DACMVA framework is assessed in a real-world biomedical domain application that includes multiple modalities, tabular data, and low-data regimes. Specifically, DACMVA is used to conduct cancer survival predictions using the publicly available dataset The Cancer Genome Atlas (TCGA) (https://www.cancer.gov/tcga), accessed on 27 December 2022. TCGA includes clinical records, omics modalities, and whole-slide imaging for over 11,000 cases comprising 33 cancer types [15]. The dataset includes either time to death or time to the last follow up. Several works have trained DL models on one or more of these modalities in order to provide robust pan-cancer predictions of survival probabilities [16–18]. We use DNA methylation (DNAm) for modality A and gene expression (mRNA) profiles for modality B.

Training a VAE to the full TCGA dataset would likely not effectively capture the complex underlying distribution using the simple Gaussian prior of the VAE. Furthermore, multiple cancers are lacking in death events, so it is not meaningful to apply the survival prediction task. To address these issues, we follow Ching et al. [16] to filter the TCGA dataset down to 10 cancers selected for having at least 50 death events, resulting in a dataset of 5250 mRNA samples and 5464 DNAm samples. The 10 cancers included are as follows: Bladder Urothelial Carcinoma (BLCA), Breast Invasive Carcinoma (BRCA), Head and Neck Squamous Cell Carcinoma (HNSC), Kidney Renal Clear Cell Carcinoma (KIRC), Brain Lower Grade Glioma (LLG), Liver Hepatocellular Carcinoma (LIHC), Lung Adenocarcinoma (LUAD), Lung Squamous Cell Carcinoma (LUSC), Ovarian Serous Cystadenocarcinoma (OV), and Stomach Adenocarcinoma (STAD).

In the present study, 10% of the dataset was used as a test set. The remaining dataset was divided between training and validation using a 90%/10% split. There were approximately 4% fewer samples of gene expression data compared to DNA methylation in the training set. Additionally, the models were evaluated in the low-data regimes of 90% and 95% missing mRNA data. The mRNA training and validation sets were reduced by these proportions. However, we conducted our evaluation on the full test set (consisting of 521 samples) for all missing percentages. Table 1 provides the number of mRNA samples in the training set for the three missing percentages.

### 2.6. Baselines

We compared the performance of the proposed DACMVA framework against the following baselines for the task of cancer survival prediction.

- Multisurv: Vale-Silva and Rohr [17]’s Multisurv model is a DL survival prediction model that demonstrates a superior performance against multiple traditional ML and deep NN models. The work investigates the prediction performance using single modalities as well as combinations of modalities. We employ their survival prediction NN architectures for gene expression data in all of our experiments, including those with augmentation. Here the non-augmented baseline is referred to as Multisurv.
- Oversampling: This method is simple oversampling with replacements from the set of synthetic B samples. We use HPs for the batch size of the synthetic samples and a weight on the loss arising from the synthetic samples.
- TDImpute: Zhou et al. [11] proposed a single AE to translate from DNA methylation to gene expression data. Their loss function aims to minimize the root mean squared error between predicted gene expression values and the true values that are paired with the input DNA methylation data. They performed a cancer survival analysis under varying percentages of missing data and outperformed traditional ML approaches. Specifically, Zhou et al. [11] conducted a robust comparison of their TDImpute method against several standard methods: synthesis using means of samples, trans-omics block missing data imputation (TOBMI), singular value decomposition (SVD), and the least absolute shrinkage and selection operator (LASSO). They evaluated both the imputation quality and the concordance index on the cancer survival prediction task using the same two modalities. Given the comprehensive analysis of baselines in the publication of TDImpute, we use TDImpute as the key baseline with which to compare DACMVA. Any performance gain of DACMVA over TDImpute would also outperform the aforementioned baselines that their research previously assessed.
- TDImpute-OV: We integrate the TDImpute architecture into our training pipeline using oversampling. That is, we first train encoder A and decoder B as in Zhou et al. [11]. Then we train the cancer prediction task by oversampling, as described in the second paragraph of Section 2.3.

### 2.7. Experiment Details

All pre-processing, data loading, and model training were conducted in Python version 3.9.7. We used the Multisurv pre-processing steps for the DNAm and mRNA modalities found on their repository, https://github.com/luisvalesilva/multisurv (accessed on 4 January 2023)[17]. Accordingly, we reduced the feature dimensions to 5000 and 1000, respectively, by filtering to the features accounting for most of the variance among the samples. The NN models for the VAEs, discriminator, and prediction task were trained using Pytorch version 1.12.0.

#### 2.7.1. VAE Training

Each layer of the encoders and decoders of the VAEs consists of a fully connected network followed by a ReLU activation. The number of neurons in each layer decreases by a factor of two from the input layer to the final hidden layer. We picked the optimal number of layers, the number of latent dimensions, and the weights on the KL loss terms by evaluating the reconstruction loss and KL loss on the validation set. Specifically, we chose HPs such that the reconstruction loss was minimized but the KL loss did not vanish. As noted by Fu et al. [13], a zero KL loss suggests that either the posterior distribution produced by the encoder is equivalent to the Gaussian prior or the decoder does not produce outputs that depend on the latent variable fed into it. For each VAE, we tuned the number of layers to be 2–7, the number of latent dimensions to be 8–512, and the weight on the KL divergence term to be 10^*−*5^–1. We settled on three layers for the encoder, three layers for the decoder, and a latent dimension of 32. We found that the optimal weight on the KL term for the modality A VAE is 0.001 and the weight for the modality B VAE is 0.0001. As previously noted, we implemented the cyclic annealing schedule put forth by Fu et al. [13] to stabilize the training.

The modality A VAE was trained first and was held fixed in subsequent steps. The modality A encoder was used to evaluate *L*_*CM*_ when training the modality B VAE. We had a weight on the loss term for the cross-modal loss *L*_*CM*_, and we found that intermediate values of 0.4–0.8 resulted in the best *L*_*CM*_ on the validation set without substantially degrading the modality B reconstruction loss.

#### 2.7.2. Adversarial Training

The CM-VAE-Adv and CM-VAE-Adv-OV methods in the DACMVA framework involve training a discriminator as well. We fixed the number of epochs to 400. We reserved the first 100 epochs to train only the modality B VAE, so that the VAE was given the chance to acquire good reconstructions before introducing adversarial training. We trained the modality B VAE with *L*_*recon*_, *L*_*KL*_, and *L*_*CM*_. After the first 100 epochs, we iterated through the following steps in each epoch. First, we trained the modality B VAE with the aforementioned losses. Then, we freezed the VAE and trained the discriminator, consisting of four fully connected layers with ReLU activations. The discriminator takes in the latent representations produced by the VAEs, and its loss function is the Least Squares GAN (LSGAN) loss [19]. To compute this loss, we assigned a label of 1 to latent representations arising from the A encoder and 0 to the latent representations arising from the B encoder. Lastly, we train the encoder of the modality B VAE, which is analogous to the generator in the adversarial AE formalism [10]. We froze the discriminator and acquired its output on the latent representations from the encoders again. We computed the LSGAN loss but with the previous labels swapped, so as to encourage the modality B encoder to fool the discriminator. Backpropagation on this loss occurred only through the modality B encoder in this step. The learning rate for the modality B VAE and the encoder was 10^*−*4^, and the learning rate for the discriminator was 5^*−*5^.

#### 2.7.3. Prediction Task Training

As in Multisurv, our task predicts the conditional survival probability for 30 years. We implemented Multisurv’s definition of *L*_*task*_ equal to the the negative log likelihood of survival in each time interval. We also borrowed Multisurv’s mRNA NN architecture for the prediction task, and we followed suit by evaluating the performance with the time-dependent concordance index (*C*_*td*_). This metric measures the model’s ability to correctly discriminate event times between patients, but it is adjusted to account for the survival function over the full prediction time window [20]. Note that Multisurv uses all 33 cancers. In contrast, the implementation here reduced the dataset to the 10 cancers mentioned above.

All experiments were trained for 400 epochs, and the model maximizing *C*_*td*_ on the validation set was saved. The learning rate was fixed to 10^*−*5^ for the prediction task in all experiments. Table 2 summarizes the experiments and the HPs associated with training the prediction task. All methods require specification of the batch size on **X**_**B**_ (bs). Methods using oversampling additionally include an HP for the batch size on ${\hat{\text{X}}}_{\text{B}}$ (bsOV). A loss threshold on *L*_*task*_ for keeping synthetic samples is dictated by an HP labeled *t*. Lastly, *γ* is the weight on *L*_*task*_ for ${\hat{\text{X}}}_{\text{B}}$ samples. Note that Table 2 pertains solely to the prediction NN and does not include the HPs associated with training the VAEs.

## 3. Results

The DACMVA framework of cross-modal variational eutoencoders (CM-VAE) with various forms of data augmentation was assessed using a real-world cancer survival prediction test using tabular data. First, the imputation quality of the DACMVA models was compared to TDImpute [11]. Next, the effect of augmentations on the prediction task in low-data regimes was assessed and compared between DACMVA models and TDImpute [11].

The experiments considered three missing percentages: 4% (the unmodified dataset), 90%, and 95%. Intermediate missing percentages such as 60% resulted in a Multisurv *C*_*td*_ of 0.683, therefore not substantially degrading the performance of the baseline. Zhou et al. [11], likewise, noted that the concordance index is not highly sensitive to missing data. Therefore, the experiments did not evaluate these intermediate missing percentages and instead focused on the unmodified dataset and the very-low-data regimes. All results provided in this section were evaluated on the full test set of 521 samples.

### 3.1. Imputation Quality

*L*_*CM*_ was evaluated on the test set. The DACMVA framework (e.g., the CM-VAE methods with and without adversarial training) performed better than TDImpute. The differences were more pronounced with higher missing data percentages, as shown in Table 3.

Differences in performance between models were assessed for statistical significance using the one-sided Wilcoxon signed-rank test to compare the DACMVA CM-VAE models (with and without adversarial training) with the TDImpute values. This test is a non-parametric evaluation of the ranked performances of two algorithms on matched pairs [21,22]. The statistical test was performed on the *L*_*CM*_ values resulting from the CM-VAE against the *L*_*CM*_ values resulting from TDImpute. The same test was conducted to compare the CM-VAE-Adv model against the TDimpute model [11]. The statistical results are shown in Table 4. All statistical comparisons resulted in *p*-values << 0.001. Thus, the experimental results indicate that the imputations produced by the DACMVA framework’s CM-VAE models (with and without adversarial training) result in *L*_*CM*_ values that are significantly better than the TDImpute model imputations.

However, a low *L*_*CM*_ does not exclude the possibility that the B decoder may have learned to produce crude averages of the training set, rather than producing a meaningful structure based on the latent input. To assess the ability of the DACMVA framework to reproduce the structure of the modality B data, Pearson’s correlation coefficient was computed between the mean feature values over the test **X**_**B**_ set and the mean feature values of the imputed ${\hat{\text{X}}}_{\text{B}}$ [23]. Let *ρ*(**X**_**B**_, ${\hat{\text{X}}}_{\text{B}}$) be the Pearson correlation coefficient function. Let *n* be the size of the set **X**_**B**_, and let $\frac{1}{n}\sum_{i}X_{B,i}$ be vector produced by averaging the *X*_*B*_ samples of the **X**_**B**_ along the feature dimension. The mean is analogously defined for ${\hat{\text{X}}}_{\text{B}}$ as well. Then, the quantity used to compare the mean feature values between the **X**_**B**_ and ${\hat{\text{X}}}_{\text{B}}$ is given as follows:

(3) $$\rho_{X_{B},{\hat{X}}_{\text{B}}}=\rho \left(\frac{1}{n}\underset{i}{\sum }X_{B,i},\frac{1}{n}\underset{i}{\sum }{\hat{X}}_{B,i}\right)$$

Additionally, we sought to understand whether samples drawn from a normal distribution and fed through the decoder maintain the structure of the **X**_**B**_ test samples. Thus, we also computed the following:

(4) $$\rho_{X_{B},Dec_{B}(𝒩(0,1))}=\rho \left(\frac{1}{n}\underset{i}{\sum }X_{B,i},\frac{1}{n_{N}}\underset{i}{\sum }Dec_{B}(𝒩(0,1))\right)$$

A large value of $\rho_{X_{B},}Dec_{B}(𝒩(0,1))$ indicates that the mean feature values produced by decoding samples from a normal distribution correlate well with the mean feature values of the original dataset. This quantity thereby provides insight into the ability of the *Dec*_*B*_ to take an input from a regularized latent space and provide an output which is structurally similar to the real samples.

Table 5 provides the Pearson correlation coefficient (PCC) for Equation (3), and Table 6 provides the PCC for Equation (4). It is shown that the DACMVA models, CM-VAE and CM-VAE-Adv, perform better than TDImpute for $\rho_{X_{\text{B}},{\hat{X}}_{\text{B}}}$, and they are substantially higher in $\rho_{X_{B},Dec_{B}}(𝒩(0,1))$ The *p*-values of the correlations are all *p* << 0.001. The results suggests that the CM-VAE and CM-VAE-Adv models create imputations that provide better structural similarities to the true dataset. The correlation improvement is particularly pronounced when drawing randomly from a normal distribution, indicating that the VAE models produce more structurally meaningful outputs when fed a sample that is very dissimilar from the training samples.

### 3.2. Effect of Augmentations in the Prediction Task

A key research question is to determine whether the data augmentations improve the performance in the downstream task. Table 7 provides *C*_*td*_ for the experiments. Due to the substantial improvement provided by OV alone, we also extended TDImpute by oversampling with replacement (TDImpute-OV). However, TDImpute-OV exhibited a degraded performance relative to TDImpute [11]. We see that the DACMVA framework of the CM-VAE variations outperforms the other experiments, and the best performing DACMVA model varies with the missing data percentage. However, the 95% confidence intervals computed from the 1000 bootstraps on the test set do overlap. As detailed in Section 3.1, a one-sided Wilcoxon signed-rank test was performed to compare the best DACMVA VAE method to TDImpute on the bootstrapped sets. The statistical results are shown in Table 8. All comparisons were very significant with *p*-values << 0.001. Therefore, it is concluded that the best DACMVA VAE method at each missing percentage (MP) significantly outperforms the previous state-of-the-art baseline, TDImpute [11].

The results indicate that adversarial training enables a substantial improvement over CM-VAE-OV in the regime of 90% fewer RNA samples than DNA samples. However, in the cases of 4% and 95%, adversarial training offers marginal or no improvement over CM-VAE-OV. We hypothesize that, in the very-low-data regime of 95%, there is insufficient data to achieve an improved latent space alignment from adversarial training. Conversely, it is possible that, at 4%, there is enough data for alignment to be achieved with *L*_*CM*_ alone. Therefore, the study results indicate that adversarial training may be beneficial for some data regimes, but it does not consistently outperform the other augmentation models.

## 4. Discussion

As part of the construction and analysis of the DACMVA framework, we experimented with multiple variations of augmentation. These variations included with or without oversampling and with or without adversarial training. Compared to TDImpute [11], the DACMVA method of training modality B as a VAE with a cross-modal loss provided an improved imputation quality, as assessed by test set *L*_*CM*_ and Pearson correlation coefficients. DACMVA also achieved an improved performance in the downstream prediction task when compared to TDImpute, oversampling alone, and the baseline without augmentation.

A noted limitation is that in the very-low-data regimes (90% and 95%), the improvement in *C*_*td*_ over TDImpute [11] was statistically significant based on a Wilcoxon signed-rank test, but the performance gain was relatively small in magnitude. Additionally, adversarial training did not consistently improve results relative to training modality B without adversarial training. The performance of the algorithms relative to one another was dependent on the percentage of missing modality B data. The performances were also sensitive to HPs. Nonetheless, the developed DACMVA framework provides a means to conduct data augmentation for the challenging scenario of tabular data modalities and continuous labels.

Future efforts will include augmenting multiple modalities via the DACMVA framework and using multi-modal inputs for the prediction task. Specifically, the inclusion of a contrastive loss, as suggested by Radhakrishnan et al. [24], could improve latent space alignment. Finally, here, the application of DACMVA focused on cancer survival prediction. It is anticipated that the performance gains will generalize to other domains and applications. Application of the DACMVA framework of models towards additional DL prediction tasks is a rich area for future research.

## Figures and Tables

**Table 1.** Number of mRNA samples (modality B) in the training set at varying missing percentages.

Cancer Type	Missing 4%	Missing 90%	Missing 95%
BLCA	322	32	17
BRCA	875	111	53
HNSC	413	47	24
KIRC	426	36	14
LLG	411	51	25
LIHC	294	35	16
LUAD	408	35	20
LUSC	396	52	26
OV	336	38	17
STAD	322	34	17
OVERALL	4203	471	229

**Table 2.** The methods and the associated HPs for training the prediction NN. Note that CM-VAE-Adv, CM-VAE-OV, and CM-VAE-Adv-OV are part of the DACMVA framework.

Method	Description	Prediction NN HPs
Multisurv	No augmentation	bs
OV	Oversample from **χ**_**B**_ with replacement	bs, bsOV, *γ*
TDImpute	Train *Enc*_*A*_, *Dec*_*B*_ as in Zhou et al. [11]; no oversampling	bs
TDImpute-OV	Train *Enc*_*A*_, *Dec*_*B*_ as in Zhou et al. [11]; with oversampling	bs, bsOV, *γ*, *t*
CM-VAE-Adv	With adversarial training; no oversampling (see Section 2.2)	bs
CM-VAE-OV	No adversarial training; with oversampling (see Section 2.3)	bs, bsOV, *γ*, *t*
CM-VAE-Adv-OV	With adversarial training; with oversampling (see Section 2.4)	bs, bsOV, *γ*, *t*

**Table 3.** Test set cross-modal loss with 95 % confidence intervals. TDImpute is compared to the DACMVA framework, namely the cross-modal variational auto-encoder (CM-VAE) and the CM-VAE with adversarial training (CM-VAE-Adv).

Missing %	TDImpute	CM-VAE	CM-VAE-Adv
4	0.0150 ± 0.0006	**0.0144** ± 0.0007	0.0146 ± 0.0006
90	0.0165 ± 0.0007	**0.0156** ± 0.0007	0.0158 ± 0.0007
95	0.0181 ± 0.0006	0.0164 ± 0.0007	**0.0160** ± 0.0007

**Table 4.** Statistical comparison of the test set’s cross-modal loss with the best DACMVA model compared to TDImpute. The best DACMVA model is shown in Table 3.

Missing %	Best DACMVA Models vs. TDImpute
4%	*p*-value = 1.2 × 10^−10^
90%	*p*-value = 6.8 × 10^−27^
95%	*p*-value = 3.3 × 10^−44^

**Table 5.** The Pearson correlation coefficient between the test set’s modality B data and samples imputed from modality A. TDImpute is compared to the DACMVA framework, namely the cross-modal variational auto-encoder (CM-VAE) and CM-VAE with adversarial training (CM-VAE-Adv).

Missing %	TDImpute	CM-VAE	CM-VAE-Adv
4	0.985	0.989	**0.992**
90	0.977	**0.989**	0.983
95	0.964	0.986	**0.987**

**Table 6.** The Pearson correlation coefficient (PCC) between the test set’s modality B data and synthetic data created by decoding normal distribution samples. TDImpute is compared to the DACMVA framework, namely the cross-modal variational auto-encoder (CM-VAE) and CM-VAE with adversarial training (CM-VAE-Adv).

Missing %	TDImpute	CM-VAE	CM-VAE-Adv
4	0.863	0.986	**0.989**
90	0.806	**0.986**	0.978
95	0.775	**0.983**	0.980

**Table 7.** Concordance Index with 95% confidence intervals from 1000 bootstraps on the test set. The DACMVA framework for cross-modal variational auto-encoders with oversampling (CM-VAE-OV), adversarial training (CM-VAE-Adv), or both (CM-VAE-Adv-OV) is compared to the other baselines in varying data regimes.

Missing Percent	Multisurv	OV	TDImpute	TDImpute-OV	CM-VAE-OV	CM-VAE-Adv	CM-VAE-Adv-OV
4	0.681 ± 0.036	0.691 ± 0.034	0.688 ± 0.035	0.667 ± 0.033	0.700 ± 0.033	0.693 ± 0.032	**0.703** ± 0.034
90	0.640 ± 0.036	0.666 ± 0.033	0.682 ± 0.033	0.675 ± 0.033	0.673 ± 0.036	**0.685** ± 0.034	0.684 ± 0.033
95	0.598 ± 0.035	0.622 ± 0.038	0.630 ± 0.035	0.621 ± 0.035	**0.639** ± 0.034	0.624 ± 0.036	0.636 ± 0.034

**Table 8.** Statistical comparison of the best DACMVA models against TDImpute on the test set with varying missing percentages. The best DACMVA model refers to the model providing the best *C*_*td*_ per Table 7. Resultant *p*-values are shown for the one-sided Wilcoxon signed-rank test.

Missing %	Best DACMVA Models vs. TDImpute
4%	*p*-value = 3.6 × 10^−155^
90%	*p*-value = 7.2 × 10^−10^
95%	*p*-value = 1.8 × 10^−82^

## Data Availability

The dataset used in this paper is publicly available from The Cancer Genome Atlas: https://www.cancer.gov/tcga (accessed on 27 December 2022). The computer code to run DACMVA is publicly available on GitHub https://github.com/srajaram24/DACMVA (accessed 16 December 2023).

## Abbreviations

The following abbreviations are used in this manuscript:

MDPI: Multidisciplinary Digital Publishing Institute
VAE: Variational Autoencoder
GAN: Generative Adversarial Network
CM: Cross-Moda
DACMVA: Data Augmentation with Cross-Modal Variational Autoencoders
TCGA: The Cancer Genome Atlas
